# *Chlamydia trachomatis dapF* Encodes a Bifunctional Enzyme Capable of Both d-Glutamate Racemase and Diaminopimelate Epimerase Activities

**DOI:** 10.1128/mBio.00204-18

**Published:** 2018-04-03

**Authors:** George Liechti, Raghuveer Singh, Patricia L. Rossi, Miranda D. Gray, Nancy E. Adams, Anthony T. Maurelli

**Affiliations:** a Department of Microbiology and Immunology, F. Edward Hébert School of Medicine, Uniformed Services University of the Health Sciences, Bethesda, Maryland, USA; b Emerging Pathogens Institute and Department of Environmental and Global Health, College of Public Health and Health Professions, University of Florida, Gainesville, Florida, USA; National Cancer Institute

**Keywords:** *Chlamydia*, diaminopimelate epimerase, evolution, glutamate racemase, moonlighting enzyme, peptidoglycan, underground metabolism, primordial enzymes

## Abstract

Peptidoglycan is a sugar/amino acid polymer unique to bacteria and essential for division and cell shape maintenance. The d-amino acids that make up its cross-linked stem peptides are not abundant in nature and must be synthesized by bacteria *de novo*. d-Glutamate is present at the second position of the pentapeptide stem and is strictly conserved in all bacterial species. In Gram-negative bacteria, d-glutamate is generated via the racemization of l-glutamate by glutamate racemase (MurI). *Chlamydia trachomatis* is the leading cause of infectious blindness and sexually transmitted bacterial infections worldwide. While its genome encodes a majority of the enzymes involved in peptidoglycan synthesis, no *murI* homologue has ever been annotated. Recent studies have revealed the presence of peptidoglycan in *C. trachomatis* and confirmed that its pentapeptide includes d-glutamate. In this study, we show that *C. trachomatis* synthesizes d-glutamate by utilizing a novel, bifunctional homologue of diaminopimelate epimerase (DapF). DapF catalyzes the final step in the synthesis of *meso*-diaminopimelate, another amino acid unique to peptidoglycan. Genetic complementation of an *Escherichia coli murI* mutant demonstrated that *Chlamydia* DapF can generate d-glutamate. Biochemical analysis showed robust activity, but unlike canonical glutamate racemases, activity was dependent on the cofactor pyridoxal phosphate. Genetic complementation, enzymatic characterization, and bioinformatic analyses indicate that chlamydial DapF shares characteristics with other promiscuous/primordial enzymes, presenting a potential mechanism for d-glutamate synthesis not only in *Chlamydia* but also numerous other genera within the *Planctomycetes*-*Verrucomicrobiae*-*Chlamydiae* superphylum that lack recognized glutamate racemases.

## IMPORTANCE

Here we describe one of the last remaining “missing” steps in peptidoglycan synthesis in pathogenic *Chlamydia* species, the synthesis of d-glutamate. We have determined that the diaminopimelate epimerase (DapF) encoded by *Chlamydia trachomatis* is capable of carrying out both the epimerization of DAP and the pyridoxal phosphate-dependent racemization of glutamate. Enzyme promiscuity is thought to be the hallmark of early microbial life on this planet, and there is currently an active debate as to whether “moonlighting enzymes” represent primordial evolutionary relics or are a product of more recent reductionist evolutionary pressures. Given the large number of *Chlamydia* species (as well as members of the *Planctomycetes*-*Verrucomicrobiae*-*Chlamydiae* superphylum) that possess DapF but lack homologues of MurI, it is likely that DapF is a primordial isomerase that functions as both racemase and epimerase in these organisms, suggesting that specialized d-glutamate racemase enzymes never evolved in these microbes.

## INTRODUCTION

The members of the family *Chlamydiaceae* are obligate intracellular bacterial pathogens that cause ocular, sexually transmitted, and respiratory infections. They exhibit a unique biphasic developmental cycle, alternating between metabolically inert elementary bodies (EBs) and metabolically active reticulate bodies (RBs) (1). Infectious EBs attach to and invade host epithelial cells and subsequently reside within a specialized host cell phagosome termed an inclusion. After invasion of a host cell, EBs differentiate into RBs that begin replicating and result in slow expansion of the inclusion. As the inclusion matures, RBs secrete effector proteins via a type III secretion system, altering the host cell trafficking machinery and resulting in the redirection of nutrients to the growing inclusion. Late in the developmental cycle, RBs differentiate back into EBs and exit the cell either by extrusion of the inclusion into the extracellular environment or as a result of host cell lysis due to uncontrolled expansion of the inclusion.

Similar to nearly all other Gram-positive and Gram-negative bacteria, pathogenic chlamydiae are susceptible to antibiotics that target peptidoglycan (2), a heteropolymer essential for bacterial cell division and maintenance of hydrostatic pressure. Numerous studies over the last several decades have failed to definitively identify peptidoglycan within chlamydiae (3, 4), despite the presence of numerous peptidoglycan synthesis genes located in the genome (4–6), giving rise to the “chlamydial anomaly” (7). Only within the last few years, thanks in large part to advances in fluorescence labeling, cryoelectron/superresolution microscopy, and direct muropeptide analysis, has convincing evidence been presented establishing that this essential cell wall component is present in members of the *Chlamydiae* (7–9).

**FIG 1:**
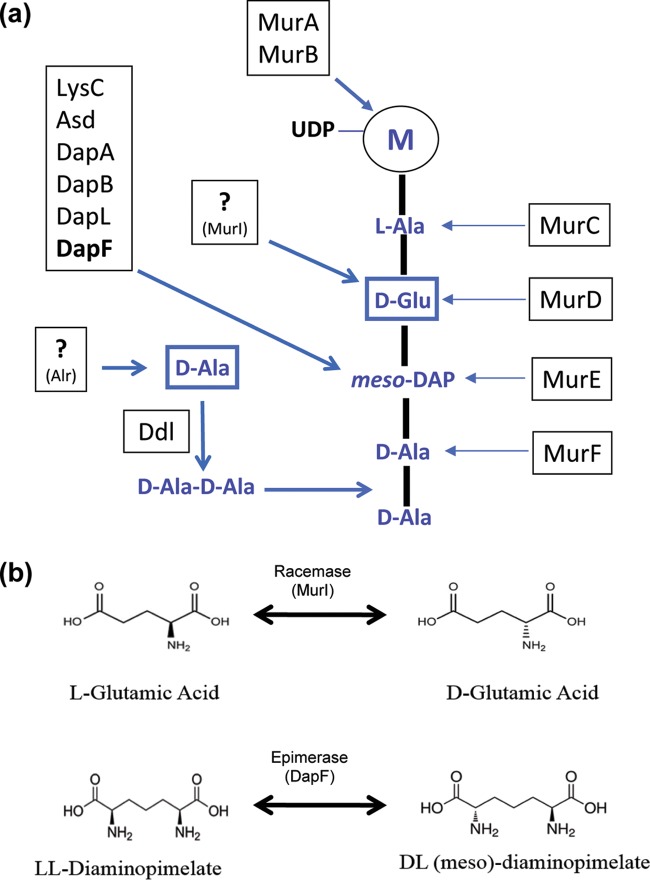
Missing enzymatic activities in the Park nucleotide biosynthesis pathway in pathogenic *Chlamydia* species. (a) Muramyl pentapeptide biosynthesis pathway in *E. coli*. Peptide synthesis is nonribosomal and instead carried out by a series of amino acid ligases (MurC, -D, -E, and -F) that serially add amino acids to newly forming peptide chains. Enzyme homologues not present in the *C. trachomatis* genome are also indicated (?). M, *N*-acetylmuramic acid. (b) Glutamate racemase versus DAP epimerase enzymatic reactions. Stereoisomers of glutamic acid and DAP. l,l-DAP, and d,l-*m*-DAP possess two amine groups, thus creating two centers of asymmetry, whereas l- and d-glutamic acids contain only a single center of asymmetry.

While a majority of the enzymes comprising the peptidoglycan biosynthetic pathway appear to be present in most members of the *Chlamydiae* (10), several key components are absent. No homologue of the gene for penicillin-binding protein 1 (Pbp1), encoding the glycosyltransferase essential for construction of the peptidoglycan glycan chains, has been identified in any of the *Chlamydiae*. Additionally, the biosynthesis pathway for generation of the Park nucleotide (the fundamental subunit of all bacterial peptidoglycan) in *Chlamydia* species is incomplete (Fig. 1a). Three noncanonical amino acids, d-alanine (d-Ala), d-glutamate (d-Glu), and *meso*-diaminopimelate (*m*-DAP), are essential for assembly of the Park nucleotide and, subsequently, peptidoglycan. As d-amino acids do not exist in abundance in nature, bacteria must either acquire them from an exogenous source or synthesize them *de novo*. The canonical pathway for the synthesis of d-amino acids involves the racemization of l-amino acids via racemase enzymes. Alternatively, specific d-amino acids can be synthesized from other d-amino acids via d-amino acid aminotransferase (Dat). Strikingly, the genomes of pathogenic *Chlamydia* species lack homologues of known racemases and d-amino acid aminotransferases (6). In most Gram-negative bacteria, d-Glu is generated via the glutamate racemase MurI (11) and is invariant in the second position of the peptidoglycan pentapeptide chain (12). All chlamydial genomes encode a UDP-*N*-acetylmuramoyl-l-alanine-d-glutamate ligase (13), which adds d-Glu to the pentapeptide chain in all of the bacteria examined to date. *murI* is an essential gene for the vast majority of bacterial pathogens (14), and while homologues are present in the genomes of various environmental chlamydiae (10), no *murI* homologue has been identified in any pathogenic *Chlamydia* species. Recent studies indicate that both pathogenic and environmental *Chlamydia* species contain *m*-DAP, d-Ala, and d-Glu in the stem peptides of their peptidoglycan (8, 9), indicating the presence of a potentially novel d-Glu synthesis pathway.

Chlamydiae are obligate intracellular organisms that grow within eukaryotic cells, and eukaryotic cells do not synthesize or maintain d-amino acids at appreciable levels (15). Therefore, in the absence of a *murI* (or *dat*) homolog, *de novo* synthesis appears to be the only method for the acquisition of d-Glu by pathogenic *Chlamydia* species. Obligate bacterial pathogens and symbionts frequently exhibit a reduced genome size due to gene loss (16, 17). Consequently, they are thought to be highly efficient in their remaining metabolic processes, although this phenomenon is not universal in all microbes (18). *Chlamydia* possesses a streamlined genome as a result of millions of years of coevolution with its vertebrate host(s), and this is indicative of a highly efficient metabolic network (19, 20). Often, “promiscuous enzymes” are relied upon by genetically reduced microbes to carry out various “moonlighting functions” otherwise performed by entirely separate proteins in other organisms (21, 22). Serine hydroxymethyltransferase (GlyA) from *Escherichia coli* is one such promiscuous enzyme in that it displays alanine racemase coactivity as a side reaction (23). *Treponema denticola* also possesses a *glyA* homologue that encodes a serine hydroxymethyltransferase with alanine racemase activity (12). A previous study indicated that *Chlamydia pneumoniae* is capable of producing d-Ala and that this activity is the direct result of its promiscuous GlyA enzyme (24).

We postulated that pathogenic chlamydiae possess a bifunctional enzyme capable of generating d-Glu for the synthesis of peptidoglycan. Racemases and epimerases both belong to the family of isomerases and are capable of altering the chirality of amino acids and their derivatives. While racemases catalyze the inversion of the configuration around an asymmetrical carbon in substrates having one center of asymmetry, epimerases perform the same function on substrates that have more than one center of asymmetry. Upon examination of the known epimerases encoded by genes in the *C. trachomatis* genome, we determined that chlamydial DapF, the DAP epimerase responsible for converting l,l-DAP to d,l-*m*-DAP (13), seemed a likely candidate for glutamate racemase activity. The structural similarity of l,l-DAP and d,l-*m*-DAP compared to l-glutamate (L-Glu) and d-Glu (Fig. 1b) led us to speculate that chlamydial DapF (DapF_CT_) may have evolved broad substrate specificity, allowing it to function as both a racemase and an epimerase in *Chlamydia*.

Here we demonstrate that DapF_CT_ is capable of racemizing l-Glu to d-Glu (and vice versa) in the presence of the cofactor pyridoxal phosphate (PLP), thereby solving the mystery of how a microbe that lacks a canonical d-Glu racemase is capable of synthesizing d-Glu-containing peptidoglycan. Interestingly, DAP and glutamate appear to be competitive substrates, indicating that they share an active site despite the racemase reaction requiring the PLP cofactor. Comparison of the predicted structure of DapF_CT_ to the solved structures of DAP epimerases encoded by other bacterial species revealed numerous amino acid changes in the enzyme’s substrate-binding site, offering a potential explanation for the relaxed substrate specificity of the chlamydial enzyme. We speculate that the reduced (i) growth rate and (ii) overall quantity of peptidoglycan synthesized by pathogenic *Chlamydia* species (as well as other members of the PVC [*Planctomycetes*-*Verrucomicrobiae*-*Chlamydiae*] superphylum) have allowed these microbes to successfully evolve for millions of years without canonical glutamate racemases. Given that the vast majority of bacteria belonging to the PVC group possess DapF homologues but lack MurI homologues, we believe that DapF_CT_ is illustrative of a primordial isomerase capable of dual racemase and epimerase activities and whose existence likely predates the emergence of specialized glutamate racemases.

## RESULTS

### *C. trachomatis dapF* restores growth in an *E. coli* d-glutamate auxotroph.

**FIG 2:**
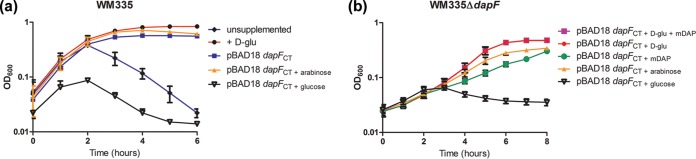
Expression of *C. trachomatis* DapF restores growth in an *E. coli* d-glutamate auxotroph. (a) Overnight cultures of strains WM335 and WM335/*dapF*_CT_ were subcultured to an OD_600_ of ~0.05 and grown in LB either unsupplemented or in the presence of d-Glu (200 µg/ml), arabinose (1%, vol/vol), or glucose (0.25%, vol/vol). OD_600_ was monitored every hour over a 6-h period. Mean values of three representative experiments are shown. (b) Overnight cultures of strain WM335Δ*dapF*/*dapF*_*CT*_ were subcultured to an OD_600_ of ~0.05 and grown in LB with thymine (50 µg/ml) under the following conditions: d-Glu (200 µg/ml), *m*-DAP (100 µg/ml) (+ mDAP), with 1% (vol/vol) arabinose, or 0.25% (vol/vol) glucose. OD_600_ was monitored every hour over an 8-h period. Data represent mean values from technical replicates of three independent biological experiments, and error bars represent the standard deviation of the mean.

To assess whether chlamydial DAP epimerase is capable of generating sufficient d-Glu to restore growth in an *E. coli* d-Glu auxotroph, we inserted a copy of the *C. trachomatis dapF* gene into expression plasmid pBAD18, placing it under the control of an arabinose-inducible promoter. We subsequently transformed this plasmid, pBAD18::*dapF*_CT_, into *E. coli* strain WM335 (25), which contains a premature stop codon in *murI* (26), as well as an essential secondary mutation in *gltS*, a d-Glu transporter (27). WM335 requires exogenous d-Glu for sustained replication, and its growth in d-Glu-free medium is highly attenuated. Once internal d-Glu stores are depleted, this strain rapidly lyses as a result of its weakened peptidoglycan layer. Cell lysis is reflected by a drop in optical density at 600 nm (OD_600_) observed after 2 h of growth in medium without d-Glu after subculture from overnight cultures (Fig. 2a). Growth curves of the pBAD18::*dapF*_CT_ transformant in the absence of exogenous d-Glu and in the presence of an inducer (arabinose) showed that expression of DapF_CT_ restored WM335 growth and continued survival when stationary phase was reached (Fig. 2a). When expression of DapF_CT_ was repressed by the addition of 0.25% glucose, no growth/survival complementation was observed.

To determine if DapF_CT_ can function as both a glutamate racemase and a DAP epimerase in a single background strain, we deleted the *E. coli dapF* allele from WM335 via lambda Red recombineering. The resulting mutant (WM335Δ*dapF*) was subsequently transformed with pBAD18::*dapF*_CT_ and analyzed for growth as before. WM335Δ*dapF* transformed with pBAD18::*dapF*_CT_ (WM335Δ*dapF*/*dapF*_CT_) exhibited slow but steady growth when induced with 1% arabinose (Fig. 2b). Upon supplementation with either d-Glu or *m*-DAP, growth complementation was achieved in the absence of the inducer. As DapF_CT_ restored WM335Δ*dapF* growth, this indicates that this enzyme appears to be capable of both DAP epimerase and d-glut racemase activities.

### Supernatants of WM335 expressing *dapF*_CT_ provide evidence of d- to l-glutamate racemization.

**FIG 3:**
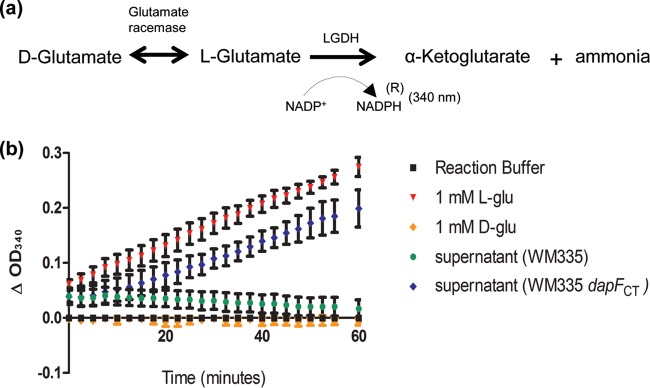
Detection of high levels of l-Glu in supernatants from overnight cultures of *E. coli* strain WM335 expressing DapF_CT_ grown with exogenous d-Glu. (a) Racemase activity was measured via a coupled biochemical reaction, i.e., the racemization of glutamate, followed by the dehydrogenation of l-Glu to α-ketoglutarate and ammonia. (b) Cultures of WM335 and WM335/pBAD18::*dapF*_CT_ were grown overnight in the presence of arabinose and exogenous d-glutamate. Supernatants were measured for the presence of l-Glu by utilizing LGDH as described in Materials and Methods. Growth medium (Luria broth) supplemented with l- or d-Glu was used for a positive or negative control (red triangles and orange diamonds, respectively). Symbols represent mean values of two independent biological replicates, and error bars represent ranges.

The standard biochemical approach for measurement of glutamate racemase activity is a coupled two-reaction assay: (i) racemization of d-Glu to l-Glu, followed by (ii) conversion of l-Glu to α-ketoglutarate subsequent to the addition of l-glutamic acid dehydrogenase (LGDH, Fig. 3a). The second reaction requires NADP as a cofactor. Conversion of l-Glu to α-ketoglutarate reduces NADP^+^ to NADPH, which is measured spectrophotometrically at 340 nm. As LGDH cannot utilize d-Glu as a substrate, production of α-ketoglutarate is indicative of glutamate racemase activity. Racemization of glutamate is reversible, and we postulated that if *C. trachomatis* DapF is capable of converting l-Glu to d-Glu, then alteration of the reaction stoichiometry should allow for the conversion of d-Glu to l-Glu in WM335 cells overexpressing DapF_CT_. To test this possibility, we assayed supernatants from overnight cultures of the parental WM335 strain and the complemented *dapF*_CT_ mutant strain, each grown in the presence of exogenous d-Glu. We found that supernatants collected from WM335 expressing DapF_CT_ contained l-Glu levels ~16-fold higher than those obtained from the parental strain (Fig. 3b), indicating that DapF_CT_ is also capable of racemizing d-Glu to l-Glu.

### *C. trachomatis* DapF possesses glutamate racemase activity that is cofactor PLP dependent.

**FIG 4:**
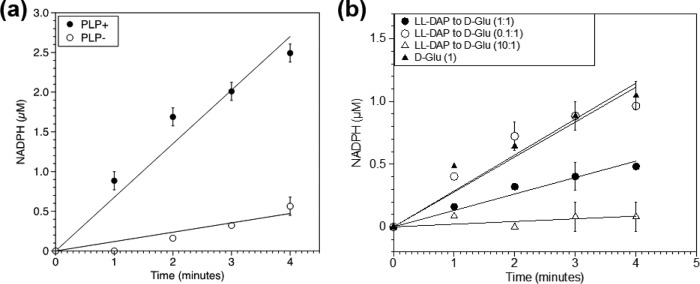
Dependence of DapF_CT_ on PLP for glutamate racemization. (a) Glutamate racemase activity of DapF_CT_ in the presence (●) or absence (○) of PLP. The glutamate racemase activity of DapF_CT_ was determined in a coupled assay as described in Materials and Methods. Error bars represent the standard deviation of two independent experiments. (b) Racemase activity of DapF_CT_ on 10 mM d-Glu alone (▲) and in the presence of 1 mM (○), 10 mM (●), or 100 mM (△) l,l-DAP. Each symbol represents the average value of two independent biological replicates.

Alanine racemases require PLP as a cofactor, whereas canonical glutamate racemases and DAP epimerases are PLP-independent enzymes that utilize a two-cysteine-dependent acid-base catalytic mechanism (15, 28). Researchers recently discovered that the cystathionine beta-lyase isolated from *Wolbachia* and *Thermotoga maritima* exhibits PLP-dependent glutamate racemase activity (29), indicating that the dual functionality of these moonlighting enzymes may require PLP as a cofactor. To test this possibility, DapF_CT_-His was overexpressed and purified for *in vitro* characterization. Resolution of the purified DapF_CT_-His protein via SDS-PAGE yielded a single band corresponding to the expected molecular mass of ~32 kDa (see Fig. S1 in the supplemental material). Purified DapF_CT_-His was assayed for glutamate racemase activity with d-Glu as the substrate as described in Materials and Methods. In the *in vitro* assay, purified DapF_CT_-His showed an increase in absorbance at 340 nm while controls remained significantly lower than the experimental reaction (Fig. S2). d-Glu racemization to l-Glu was found to be PLP dependent (Fig. 4a).

10.1128/mBio.00204-18.1 FIG S1  SDS-PAGE analysis of various eluted fractions of purified DapF_CT_-His expressed in *E. coli* BL21. Lanes: 1, molecular mass markers; 2 to 4, elution of DapF_CT_-His; 5, whole-cell lysate of *E. coli* BL21 expressing DapF_CT_-His. The protein corresponding to cloned DapF_CT_-His is boxed. Download FIG S1, TIF file, 11 MB. Copyright © 2018 Liechti et al. 2018 Liechti et al. This content is distributed under the terms of the Creative Commons Attribution 4.0 International license.

10.1128/mBio.00204-18.2 FIG S2  Assay of DapF_CT_ for glutamate racemase activity with the glutamate racemase-coupled assay. Reaction mixtures were prepared with 20 mM d-glutamate (●), without DapF (○), and without d-glutamate (△). An increase in NADPH formation is indicative of glutamate racemase activity of DapF_CT_. Error bars represent the standard deviations of two biological replicates. Download FIG S2, TIF file, 13.6 MB. Copyright © 2018 Liechti et al. 2018 Liechti et al. This content is distributed under the terms of the Creative Commons Attribution 4.0 International license.

As PLP-dependent and PLP-independent isomerases are thought to operate by dissimilar mechanisms, it was not entirely clear whether DapF_CT_ utilized the same substrate-binding pocket to carry out both racemase and epimerase reactions. To assess whether l,l-DAP and l-Glu compete for the same substrate-binding/active sites on DapF_CT_, competition assays were performed. The addition of 10 and 100 mM l,l-DAP to the reaction mixture decreased glutamate racemase activity 47 and 95%, respectively, while 1 mM l,l-DAP did not appear to substantially inhibit the reaction (Fig. 4b). These data indicate that both substrates appear to have a binding site on DapF_CT_, that this site is occupied by either d-Glu or l,l-DAP in a concentration-dependent manner, and that the efficiency of glutamate racemization is inversely proportional to the amount of competing l,l-DAP present.

### Structure of *Chlamydia* DAP epimerase exhibits significant remodeling in the substrate-binding pocket.

**FIG 5:**
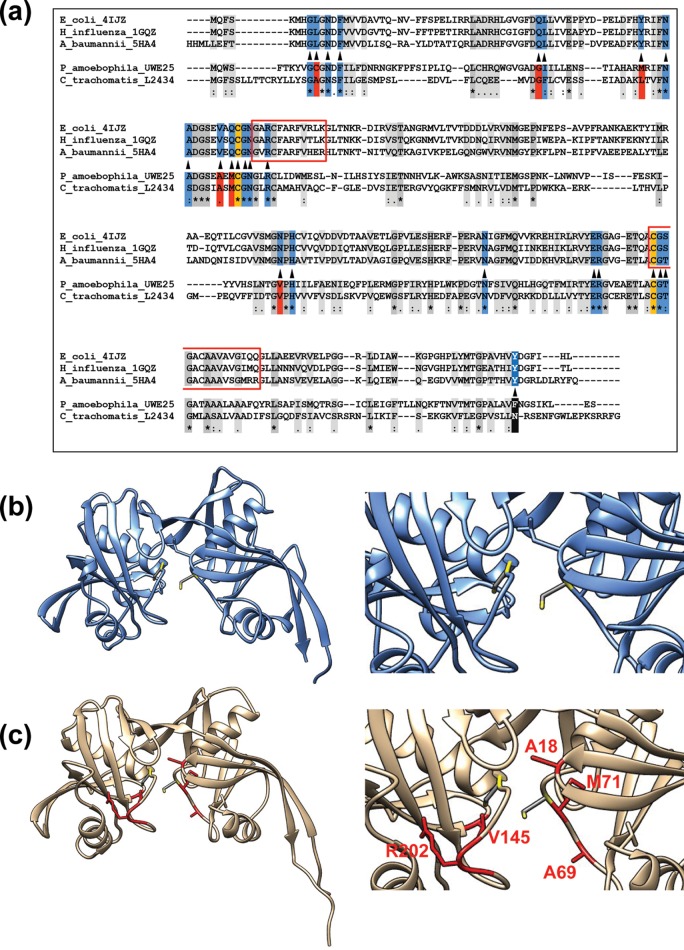
*C. trachomatis* DAP epimerase exhibits significant remodeling of the enzyme substrate-binding pocket. (a) Amino acid structural alignments indicate that of the 18 to 20 generally well-conserved amino acid residues lining the active site within the enzyme substrate-binding pocket (blue), 6 are highly dissimilar in chlamydial DapF (red). Boxed residues indicate alpha-helical domains critical for determination of the distance between active-site cysteine residues C72 and C207 (gold). DAP epimerase encoded by *B. anthracis* (b) is the enzyme most structurally similar to the DAP epimerase encoded by *C. trachomatis* present in the PDB (2OTN) and was used as the template for the modeling of a structural approximation of DapF_CT_ (c). Active-site cysteine residues (yellow) are conserved, as is their orientation within the active site, because of the presence of similar alpha-helical domains (a, boxed in red). Amino acid alterations within the binding pocket (A18, A69, M71, V145, and R202) are red, and the chlamydial enzyme’s active-site cysteine residues (C72 and C207) are yellow.

As DapF_CT_ appears to make use of the same substrate-binding pocket when carrying out both epimerase and racemase activities, we reasoned that the enzyme’s active site likely possesses unique amino acid substitutions compared to the more specialized DAP epimerases encoded by other bacterial species. In a majority of the DAP epimerases whose crystal structures have been resolved, the amino acids lining the active-site cavity are highly conserved (15, 30, 31) (Fig. 5a). In the DAP epimerases encoded by *Haemophilus influenzae* and *Bacillus anthracis*, the active-site residues are flanked by two internal α-helixes cradled by β-sheets. These helices are positioned end-on toward each other (Fig. 5b), and their orientation and lengths are crucial to the relative proximity of two active-site cysteine residues (30, 31). One active-site cysteine thiolate acts as a base to deprotonate the α-carbon, while a second cysteine thiol acts as an acid to reprotonate the resulting planar carbanionic intermediate from the opposite face (13). To access the level of conservation of the DapF_CT_ active site, we sorted proteins present in the Protein Data Bank (PDB) for enzymes that were most similar to DapF_CT_ on the basis of structural alignments of amino acid sequences (paired with solved PDB structures). The DAP epimerase encoded by *B. anthracis* (2OTN) was the most structurally similar to the *Chlamydia* enzyme (as determined by RaptorX and I-TASSER structural prediction web-based applications) and was used as a template for modeling of a structural approximation of DapF_CT_ (Fig. 5b and c). Both active-site cysteines (C72 and C207) are conserved in the chlamydial enzyme, as are the histidine and glutamic acid residues (H147 and E197) that are essential for epimerase catalytic activity (30). Of the 18 remaining amino acids that reside within the enzyme active site, only 9 (G17, N20, F22, N63, G73, N74, N179, R198, and G208) are well conserved in the *C. trachomatis* protein (Fig. 5a). Of the remaining nine amino acids, three are replaced with similar amino acids (F46, S64, T209) and six represent dissimilar amino acid substitutions (A18, G45, L59, V83, Q85, V145). We hypothesize that amino acid alterations within the binding pocket (A18, A69, M71, V145, and R202) likely influence the accessibility of substrates to the chlamydial enzyme’s active-site cysteine residues (C72 and C207).

### Chlamydial DapF racemase activity requires active-site cysteines.

**FIG 6:**
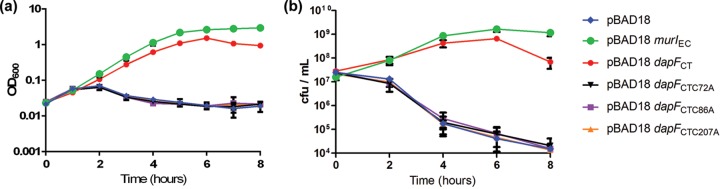
Chlamydial DapF requires the epimerase active-site cysteines for racemase activity. Mutagenesis of cysteine resides within the predicted active site of chlamydial DapF results in loss of growth/survival complementation of WM335. Cultures were grown overnight in LB supplemented with ampicillin, thymine, and d-Glu. Overnight cultures were subcultured to an OD_600_ of 0.025 in LB supplemented with ampicillin, thymine, and arabinose (1%, vol/vol). Cultures were then grown with shaking at 37°C, and the OD_600_ (a) was recorded hourly for 8 h. To determine the number of CFU/ml (b), bacterial samples taken every 2 h were plated on LB agar containing ampicillin, thymine, and d-Glu. Complementation of WM335 with *E. coli murI* was included as a positive control (green). The data are mean values of technical replicates of three independent biological experiments, and error bars represent the standard deviation of the mean.

As mentioned previously, both canonical d-Glu racemase and DAP epimerase are PLP-independent enzymes and utilize a two-cysteine-dependent acid-base catalytic mechanism (32). Sequence alignments revealed that the two active-site cysteines for epimerase function are conserved in DapF_CT_ (Fig. 5a). As our enzymatic assays established that the glutamate racemase activity of DapF_CT_ is PLP dependent, we questioned whether these active-site cysteines play any role in this activity. We mutated each of the two putative active-site cysteines in DapF_CT_ and assessed the mutated enzyme’s ability to complement the d-Glu auxotrophy of the WM335 parental strain. Interestingly, neither of these mutated alleles (C72A or C207A) was capable of restoring WM335 growth in the absence of exogenous d-Glu (Fig. 6), indicating that, despite being a PLP-dependent reaction, the racemase activity of DapF_CT_ appears to be dependent on both active-site cysteine residues. DapF_CT_ possesses an additional cysteine (C86) that is not highly conserved in other bacterial species (Fig. 5a). This cysteine sits at the terminus of one of the two predicted alpha-helical domains (opposite C72), which together determine the distance between the enzyme’s active-site cysteines and thus the functionality of the enzyme. A C86A substitution also resulted in loss of the ability of DapF_CT_ to restore growth (Fig. 6), suggesting that despite requiring PLP as a cofactor for the racemization of glutamate, DapF_CT_ requires the epimerase active-site cysteines for glutamate racemase activity.

### Phylogenetic analysis of *Chlamydia* DapF shows high similarity to DapF in other members of the PVC group superphylum.

**FIG 7:**
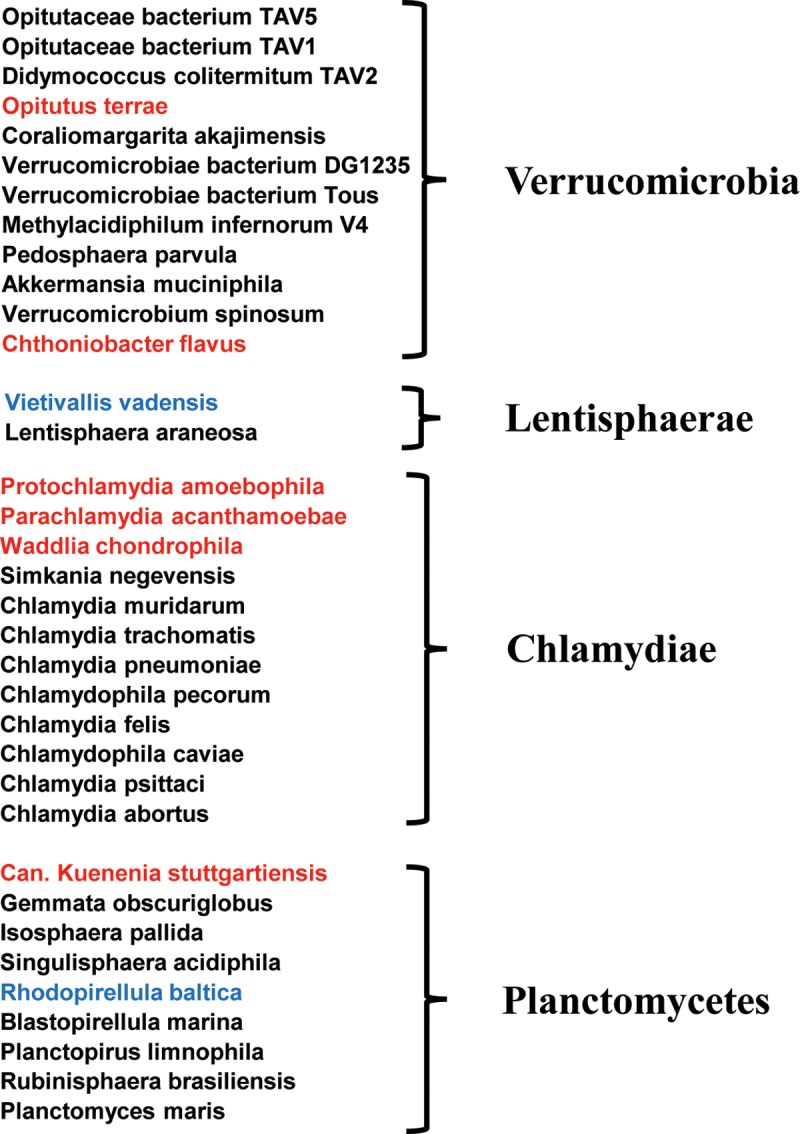
Absence of MurI homologues is prevalent in microbes of the PVC group superphylum. Shown is a list of representative species comprising the PVC group superphylum. Species possessing homologues of the DAP epimerase encoded by *P. acanthamoebae* (DapF_PA_) but lacking homologues of the glutamate racemase (MurI_PA_) are black. Species lacking both DapF_PA_ and MurI_PA_ homologues are blue. Species encoding homologues of both DapF_PA_ and MurI_PA_ are red. More comprehensive lists of PVC group members arranged by DAP epimerase/glutamate racemase status are provided in Tables S3 and S4.

As DapF_CT_ has glutamate racemase activity, we deduced that DAP epimerases might function similarly in other bacterial species closely related to *C. trachomatis*. DAP epimerases encoded by pathogenic *Chlamydia* species all share moderate sequence similarity (>48% identity, >65% similarity) and all lack *murI* homologues, suggesting that their DapF enzymes are also capable of racemizing glutamate. We questioned whether similarity to the *C. trachomatis* DapF enzyme significantly correlated with the absence of homologues of *murI* in other members of the PVC superphylum. Using protein sequences from *Parachlamydia acanthamoebae*, a chlamydia-like endosymbiont of amoebae that possesses both DapF and MurI homologues, we screened members of the PVC group for the presence/absence of these two enzymes. For the PVC group members on the NCBI database at the time of analysis (PVC group: taxid: 1783257, 5 December 2017), we performed BLAST searches for homologues of *P. acanthamoebae* 50S ribosomal protein L3, MurA (initiates the first committed step in peptidoglycan synthesis), DapF (DAP epimerase), Alr (alanine racemase), and MurI (glutamate racemase). The analysis produced affirmative alignment scores (>e^−4^) for 396, 367, 270, and 174 enzyme homologues, respectively. These numbers did not vary significantly when the enzyme sequences of other representative species were BLAST searched against the PVC group taxonomic identity (Table S1), minimizing the possibility that potential MurI homologues were missed in the initial BLAST search because of divergence from the *P. acanthamoebae* homologue. Additionally, the 174 examples of PVC group members that possess a MurI enzyme is likely an overestimate of potential d-Glu racemase homologues; several of these “positive” hits were assigned to *C. trachomatis* and *C. abortus* isolates. Upon closer inspection, the MurI homologues assigned to these isolates have low sequence similarity to each other (between species) and high sequence similarity to commensal/pathogenic microbes known to share the mammalian reproductive/vaginal niche (Table S2). This analysis further indicates that the absence of a specialized glutamate racemase is highly prevalent among members of the PVC group (Fig. 7).

10.1128/mBio.00204-18.3 TABLE S1  Glutamate racemase homologues of enzymes encoded by different PVC group members resulting from pBLAST searches. Download TABLE S1, XLSX file, 0.01 MB. Copyright © 2018 Liechti et al. 2018 Liechti et al. This content is distributed under the terms of the Creative Commons Attribution 4.0 International license.

10.1128/mBio.00204-18.4 TABLE S2  Characterization of “glutamate racemase” homologues assigned to pathogenic *Chlamydia* species. Download TABLE S2, XLSX file, 0.01 MB. Copyright © 2018 Liechti et al. 2018 Liechti et al. This content is distributed under the terms of the Creative Commons Attribution 4.0 International license.

10.1128/mBio.00204-18.5 TABLE S3  PVC group members possessing DAP epimerase (DapF) and lacking d-glutamate racemase (MurI) homologues. Download TABLE S3, XLSX file, 0.02 MB. Copyright © 2018 Liechti et al. 2018 Liechti et al. This content is distributed under the terms of the Creative Commons Attribution 4.0 International license.

10.1128/mBio.00204-18.6 TABLE S4  PVC group members possessing both DAP epimerase (DapF) and d-glutamate racemase (MurI) homologues. Download TABLE S4, XLSX file, 0.02 MB. Copyright © 2018 Liechti et al. 2018 Liechti et al. This content is distributed under the terms of the Creative Commons Attribution 4.0 International license.

## DISCUSSION

Every chlamydial genome sequenced to date encodes a nearly complete peptidoglycan synthesis pathway, with the exception of two key genes, *murI* and *alr*, which encode glutamate and alanine racemases, respectively (6). We hypothesized that the structural similarity between l-Glu and l,l-DAP makes DapF a good candidate for *Chlamydia*’s “missing” glutamate racemase. Our data indicate that *C. trachomatis* DapF can complement the d-Glu auxotrophy of an *E. coli* mutant that expresses a nonfunctional glutamate racemase. Biochemical assays confirmed that chlamydial DapF possesses glutamate racemase activity.

These findings, while solving one long-standing mystery of chlamydial cell wall biology, also raise several questions regarding the functionality of this enzyme and its potential regulation by substrate availability. Glutamate racemization is a PLP-independent mechanism in most model organisms; however, another example of PLP-dependent glutamate racemase activity has recently been reported (29). While this study is the first to demonstrate glutamate racemase activity of a DapF protein, surprisingly, this activity is PLP dependent. Paradoxically, the two active-site cysteine residues are not thought to play any role in PLP-dependent racemase activity (29), and yet our data suggest that they are essential for DapF_CT_ to complement growth in an *E. coli* d-Glu auxotroph. While epimerase active-site cysteines have been replaced in other systems without significantly affecting protein expression/integrity (33, 34), we cannot rule out the possibility that, in addition to being essential for epimerase function, these cysteine residues may also be indispensable for the overall structural stability of the *Chlamydia* enzyme. Regardless, as both substrates likely compete for the same substrate-binding pocket, it appears that *Chlamydia* has sacrificed enhanced efficiency to maintain the dual functionality of this enzyme. DapF_CT_ is capable of racemizing glutamate while maintaining enzyme kinetics similar to those of MurI_EC_; however, this high rate of activity is only possible at low levels of exogenous l,l-DAP (Fig. 4b). It is possible that the presence of competing substrates or availability of the PLP cofactor plays a role in the favoring of one reaction over the other *in vivo*. Further studies are needed to assess if and how these competing reactions are regulated, how *Chlamydia* maintains the balance between competing substrates, and how reliance on this single enzyme for the construction of two essential building blocks of the bacterial cell wall affects *Chlamydia* growth and development.

The frequency of promiscuous enzymes is generally thought to be a function of genome size and the underlying redundancy often seen in larger bacterial genomes (35). Mutations in extra copies of a given allele give rise to novel enzymes with new substrates and activities (36). This is exemplified in the patchwork model of enzyme evolution in which enzymatic specialization increases over time as a result of gene duplication and divergence (35). This model, currently favored for modern enzyme evolution, is based on the underlying assumptions that increased enzymatic efficiency is selectively advantageous and that this pressure for faster reactions results in a narrowing of enzyme function and substrate specificity. While this may be the case for a majority of the bacterial species studied to date, these efficiencies may confer less of a benefit, or may even be detrimental, in slow-growing microbes that live in relatively stable, nutrient-rich environments (such as pathogenic *Chlamydia* species and potentially other microbes comprising the PVC superphylum).

D’Ari and Casadesus coined the term “underground metabolism” to describe reactions that occur when enzymes act on substrate analogues that are also endogenous metabolites utilized by an organism (37). They argued that a certain level of metabolic inaccuracy is actually energetically efficient and that the resulting metabolic plasticity may provide an evolutionary advantage in some environments. In the case of DapF_CT_, substrate diversity appears to have been selected for as both substrates (d-Glu and *m*-DAP) are useful to the cell. DapF is not the only enzyme in the *Chlamydia* peptidoglycan biosynthetic pathway that exhibits substrate promiscuity; *C. trachomatis* UDP-*N*-acetylmuramate-l-alanine ligase (MurC) incorporates glycine, as well as l-alanine, into the first position on the peptidoglycan stem peptide (9, 38, 39). It is likely that in environments where enhanced metabolic rates are not required (or are potentially even selected against), the pressure for enzymatic efficiency is relaxed, and the activities of promiscuous enzymes can be maintained or returned to a more primordial state. Given the number of PVC group members lacking MurI homologues and encoding DapF enzymes with high similarity to DapF_CT_, it is likely that in this case a primordial isomerase with dual substrate specificity was maintained by numerous species within this superphylum. Promiscuous enzymes have recently been identified in a variety of other bacteria with reduced genomes (29) and likely play an essential role in the adaptive strategies associated with genomic/metabolic streamlining.

Numerous other bacteria outside the PVC superphylum, including *Rickettsia*, *Xanthomonas*, *Xylella*, and *Bordetella* species, also contain a nearly complete peptidoglycan biosynthesis pathway, lack a MurI homologue, and retain a DAP epimerase. It is tempting to speculate that the DAP epimerases encoded by these diverse species also possess glutamate racemase activity. Furthermore, the genomes of chlamydiae contain a high number of plant-like genes whose products are targeted to the chloroplast, suggesting an evolutionary relationship between chloroplasts and chlamydiae (40). The DAP synthesis pathway is unique in pathogenic *Chlamydia* species; these microbes lack *dapD*, *dapC*, and *dapE* homologues in their genomes and instead produce DAP by an aminotransferase thought previously to be utilized only by plants and cyanobacteria (13). This aminotransferase enzyme (DapL) also exhibits substrate promiscuity; however, its active-site resides are largely conserved with those of homologous enzymes in other bacteria (41). The genome of the nonseed moss *Physcomitrella patens* contains 10 genes involved in peptidoglycan synthesis, with several of those genes, including *murE* and *pbps*, encoding functional proteins. Moreover, *P. patens* is sensitive to β-lactam antibiotics, fosfomycin, and d-cycloserine (42–44) and incorporates d-amino acids (45), indicating that the plastid contains peptidoglycan. While a *murI* homologue has not been identified in the *P. patents* genome, it does encode a homologue of DAP epimerase. Should this enzyme also function as a glutamate racemase, it would further the evolutionary link between *Chlamydia* and chloroplasts and provide insight into how primordial microbial systems met their fundamental growth requirements with genomic efficiency and enzymatic versatility. Additionally, *Chlamydia* species lack the final enzyme in the lysine biosynthesis pathway (DAP decarboxylase, LysA), indicating that the sole purpose of *m*-DAP production by the microbe is peptidoglycan biosynthesis. It is worth noting that *Chlamydia* possesses numerous other examples of metabolic pathways apparently lacking critical enzymes essential for their ultimate functionality (22). More examples of promiscuous, primordial enzymes likely remain hidden within its efficiently streamlined genome, waiting to be discovered.

## MATERIALS AND METHODS

### Bacterial strains, medium, and growth conditions.

The *E. coli* WM335 *dapF* double mutant was constructed by lambda Red recombination (46) with WM335 (25) as the parent strain. All strains were grown in LB medium with aeration or on agar plates. The medium was supplemented with ampicillin (100 μg/ml), chloramphenicol (10 μg/ml), thymine (50 μg/ml), d-glutamic acid (200 μg/ml), arabinose (1%), glucose (0.25%), and DAP (racemic mixture; 100 μg/ml; Sigma, St. Louis, MO) as needed, and cultures were incubated at 37°C.

### Cloning, *in vivo* complementation, and recombinant expression of DapF from *C. trachomatis*.

*dapF* of *C. trachomatis* and *murI* of *E. coli* were cloned into expression vector pBAD for complementation of the *murI* mutant of *E. coli*. To express and purify DapF_CT_, *dapF*_CT_ was cloned into pTBSG (47) with a 6-His tag placed at the 5′ end of *dapF*_CT_. pTBSG::*dapF*_CT_L2 was graciously provided by Scott Hefty (University of Kansas). This plasmid was expressed in *E. coli* BL21(DE3) at small and large scales. Initially, this strain was grown at 37°C and slower induction at 16°C was done at an OD_600_ of 0.45 with a final isopropyl-β-d-thiogalactopyranoside (IPTG) concentration of 0.5 mM. Cells were harvested after 12 h of growth and resuspended in 20 mM Tris-Cl (pH 8.0)–500 mM NaCl–20 mM imidazole. Cells were lysed by incubation with lysozyme for 30 min on ice and then sonicated with an Ultrasonic Processor. A cell extract was obtained after separation of cell debris by centrifugation (10,000 × *g* for 15 min at 4°C). The soluble lysate was applied to a Ni-nitrilotriacetic acid column. After the column was washed with washing buffer (20 mM Tris-Cl [pH 8.0], 500 mM NaCl, 20 mM imidazole), the His-tagged DapF protein was eluted from the column with elution buffer (20 mM Tris-Cl [pH 8.0], 500 mM NaCl, 300 mM imidazole). All protein fractions were analyzed by SDS-PAGE. Fractions containing the greatest abundance of His-tagged DapF_CT_ were pooled and dialyzed with a dialysis tube with a 10-kDa molecular mass cutoff (Pierce Biotechnology, Waltham, MA) against storage buffer that contained 20 mM Tris-Cl (pH 7.7) and 150 mM NaCl. The His tag was not removed, as the His-tagged version of DapF complemented the *murI* mutant and the purified enzyme with or without the tag showed no significant difference in enzymatic activity (data not shown).

### Site-directed mutagenesis.

Overlapping oligonucleotides were used to introduce the desired site-directed mutations via PCR. NEA292 (5′-CAATGGCGCACGTAGCCCAG**GC**CTTTGGACTTGAAGATGTTTC-3′) and NEA293 (5′-GAAACATCTTCAAGTCCAAAG**GC**CTGGGCTACGTGCGCCATTG-3′) or NEA294 (5′-GTGAGCGAGAAACCTTATCT**GC**TGGGACAGGGATGTTGGCAAG-3′) and NEA295 (5′-CTTGCCAACATCCCTGTCCCA**GC**AGATAAGGTTTCTCGCTCAC-3′) were used to amplify *dapF*_CT_ with a C86A or C207A mutation, respectively, by using PfuUltra HF polymerase (Stratagene; La Jolla, CA). The boldface base pairs are the changed nucleotides. The PCR was treated with DpnI and transformed into DH5α competent cells. Plasmids from the resulting ampicillin-resistant colonies were sequence verified for the desired site-directed mutations.

### Growth curves.

Growth curves were determined by diluting overnight cultures into Difco LB Miller (Luria-Bertani) broth base (Becton, Dickinson and Company, NJ) with appropriate antibiotics and supplements as required to an initial OD_600_ of either 0.025 or 0.05. Cultures were then grown with shaking at 37°C for 6 to 8 h with OD_600_ readings taken every hour. To ensure that changes in OD_600_ values correlated with bacterial numbers, samples were taken every 2 h, bacteria were serially diluted and plated on LB agar, and CFU counts were determined.

### Enzyme assays.

The generation of l-Glu from d-Glu in *E. coli* expressing chlamydial DapF was measured by an enzyme-coupled reaction. Supernatants from *E. coli* grown overnight in the presence of exogenous d-Glu (200 μg/ml) were passed through a 0.22-μm filter. Ten microliters was then added to assay buffer (2.5 mM NADP^+^) containing 3 μl of l-glutamate dehydrogenase (LGDH) from bovine liver (Sigma), which catalyzes the conversion of l-Glu to α-ketoglutarate. The increase in absorbance at 340 nm (which indicates the reduction of NADP^+^ to NADPH) was measured in a BioTek Synergy 2 plate reader at 25°C, and measurements were taken every 2.5 min for an hour. For the purified, His-tagged DapF_CT_ protein, the reaction mixture with a final volume of 0.1 ml contained 20 mM Tris-Cl (pH 8.0), 20 mM d-Glu, 10-μM PLP, 10 mM MgCl_2_, and 20 μg of purified, His-tagged DapF_CT_. Reaction mixtures were incubated at 37°C for 1 h and then heat inactivated at 95°C prior to the l-Glu assay. The l-Glu assay reaction mixture with a final volume of 0.1 ml contained 20 mM Tris-Cl (pH 8.0), 2.5 mM NADP^+^, 10 mM MgCl_2_, and 50 μl of the first reaction mixture (d-Glu→l-Glu). The OD_340_ was noted immediately after the addition of 2.5 μl of LGDH. Each reaction was performed in biological replicates. To test the substrate affinity of DapF_CT_ and assess the effects of the competing substrate on the glutamate racemization reaction, various concentrations of l,l-DAP (1 to 100 mM) were added to the reaction mixture while the concentration of d-Glu was kept constant (10 mM). The resulting glutamate racemase activity (in the presence or absence of l,l-DAP) was used to describe the relationships between competing substrates (d-Glu and l,l-DAP).

### Bioinformatics.

Sequence alignments and protein structural prediction were conducted with RaptorX (48) and I-TASSER (49) web-based applications. Mapping of amino acids and visualization of DapF_CT_ structural predictions were accomplished with the UCSF Chimera imaging, analysis, and visualization suite (50).
