# Supplementary figures and images for: Chlamydia trachomatis dapF Encodes a Bifunctional Enzyme Capable of Both d-Glutamate Racemase and Diaminopimelate Epimerase Activities
Source: mBio. 2018 Apr 3;9(2):e00204-18. doi: 10.1128/mBio.00204-18 (PMC5885031; doi:10.1128/mBio.00204-18)

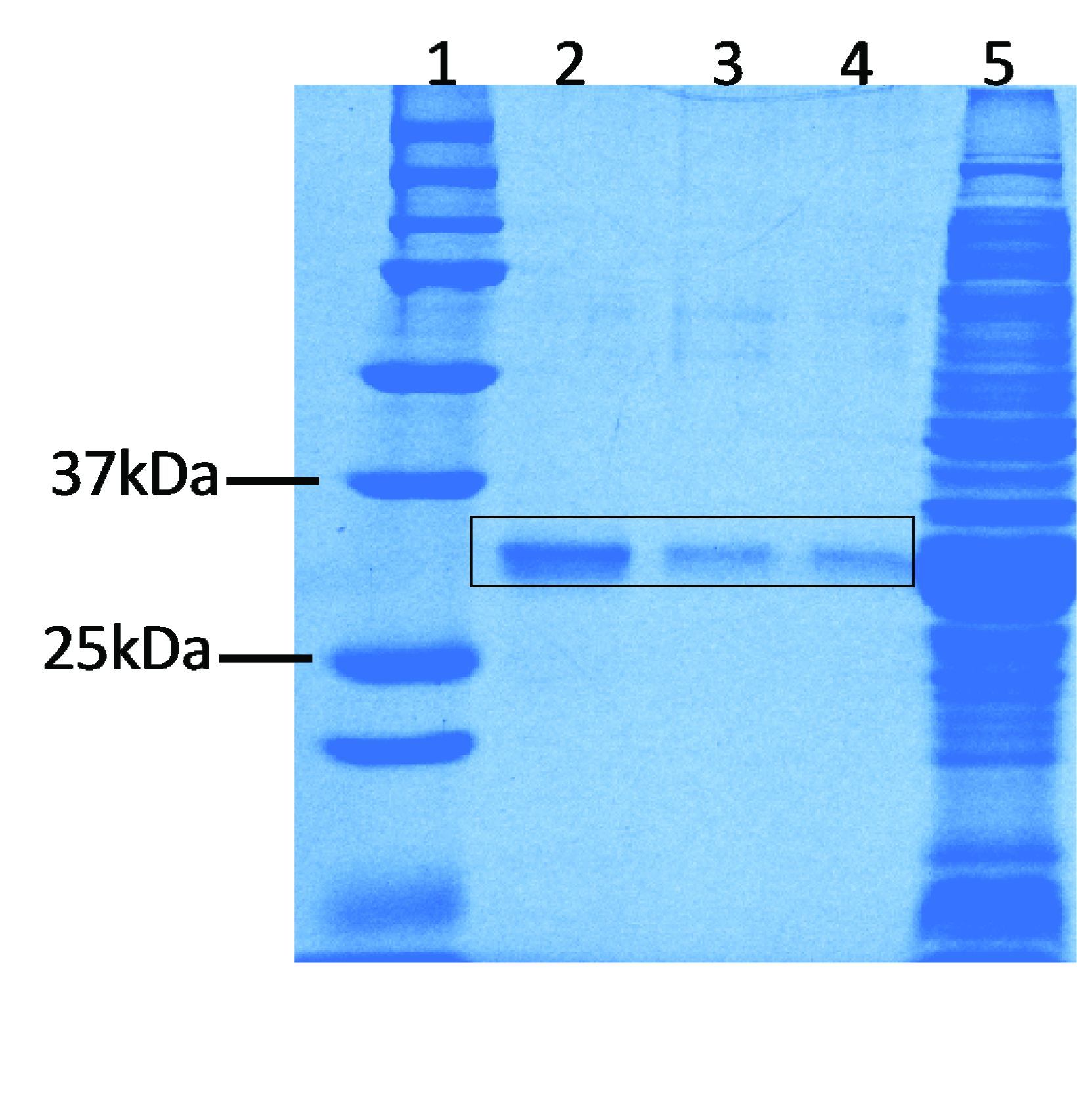

Supplement: FIG S1 [file mbo002183785sf1.tif]

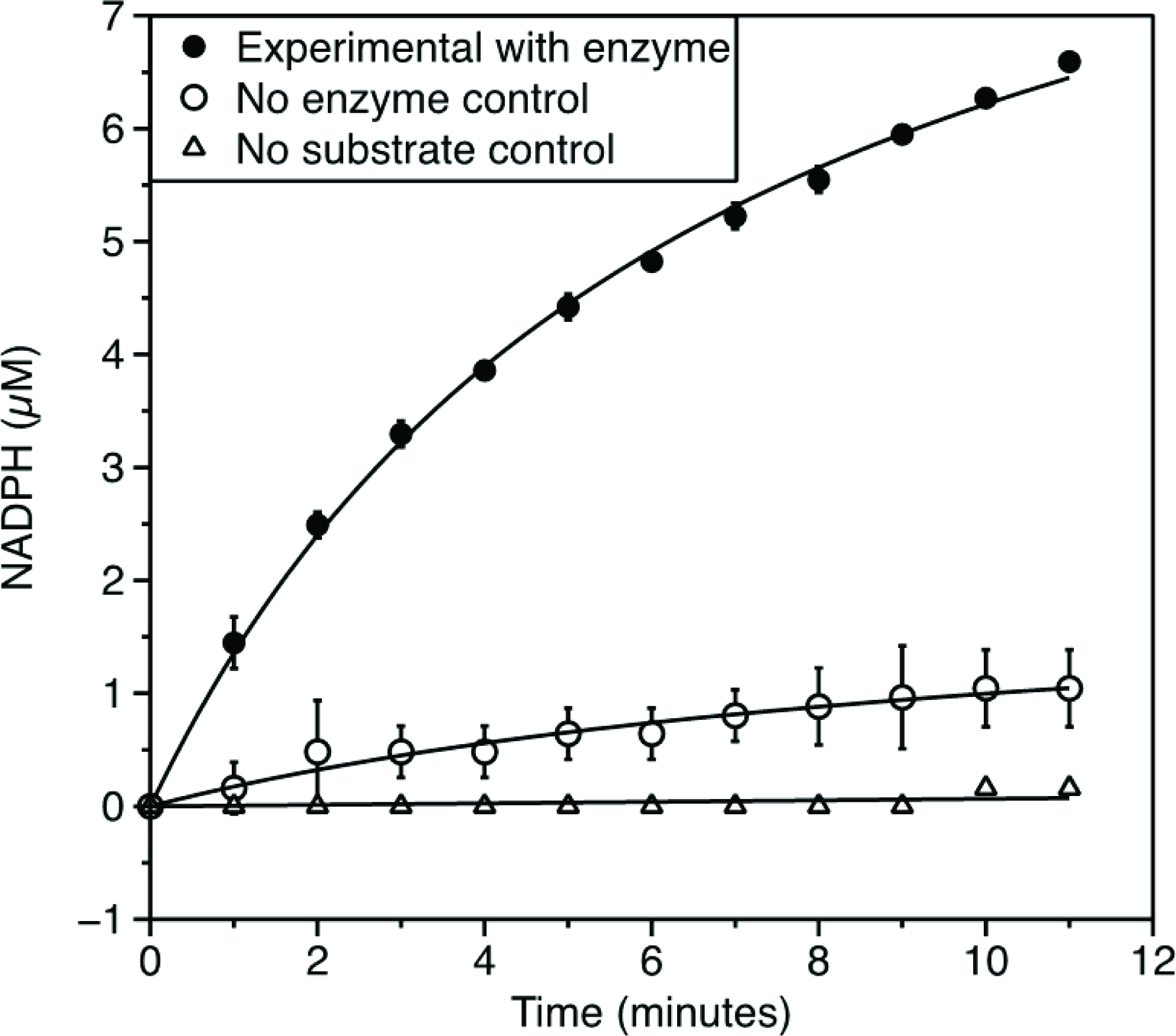

Supplement: FIG S2 [file mbo002183785sf2.tif]
